# Real-World Before–After Study of *Gastrodia elata Blume*-Based Nutraceutical Supplementation in Patients with Radiculopathy and Carpal Tunnel Syndrome

**DOI:** 10.3390/nu18091438

**Published:** 2026-04-30

**Authors:** Marco Invernizzi, Lorenzo Lippi, Simone Mulè, Rebecca Galla, Arianna Folli, Domenico Tiso, Francesca Parini, Francesca Uberti

**Affiliations:** 1 Department of Health Sciences, University of Piemonte Orientale (UPO), 28100 Novara, Italy; marco.invernizzi@med.uniupo.it (M.I.); lorenzo.lippi@uniupo.it (L.L.); arianna.folli23@gmail.com (A.F.); 2 Translational Medicine, Dipartimento Attività Integrate Ricerca e Innovazione (DAIRI), Azienda Ospedaliero-Universitaria (AOU) SS, Antonio e Biagio e Cesare Arrigo, 15121 Alessandria, Italy; 3 Department for Sustainable Development and Ecological Transition, University of Piemonte Orientale (UPO), Piazza Sant’Eusebio 5, 13100 Vercelli, Italy; simone.mule@uniupo.it; 4 Noivita Srls, Spin-Off University of Piemonte Orientale, Strada Privata Curti 7, 28100 Novara, Italy; rebecca.galla@noivita.it (R.G.); francescaparini00@gmail.com (F.P.); 5 Department of Clinical Nutrition, “Villa Maria” Hospital, 47921 Rimini, Italy; dottortiso@gmail.com

**Keywords:** nutraceutical supplementation, *Gastrodia elata Blume*, radiculopathy, carpal tunnel syndrome, functional outcomes, real-world clinical study

## Abstract

**Background/Objectives**: Cervical and lumbar radiculopathy and carpal tunnel syndrome (CTS) are common conditions that are associated with persistent pain, functional impairment, and reduced quality of life. Nutraceutical strategies targeting neuroinflammatory and oxidative stress pathways are being investigated as adjunctive approaches in pain management. This study evaluated the clinical association between supplementation with a *Gastrodiae elata Blume*-based nutraceutical formulation (Assonal^®^) and changes in pain intensity, functional outcomes, symptom burden, and quality of life in patients with radiculopathy and CTS. **Methods**: A single-centre pragmatic before–after clinical study enrolled adults with cervical or lumbar radiculopathy and/or CTS. Participants received Assonal^®^ (two tablets daily) for two months. Pain intensity was assessed using the Numerical Pain Rating Scale (NPRS) and Visual Analogue Scale (VAS). Secondary outcomes included the Back Pain Functional Scale (BPFS), the Boston Carpal Tunnel Questionnaire (BCTQ), the Neuropathic Pain Symptom Inventory (NPSI), the EuroQol-5D-5L (EQ-5D-5L), and the Global Perceived Effect (GPE). Longitudinal within-subject changes were analysed using repeated-measures statistical tests. **Results**: Thirty-four participants completed the study. The observed pain intensity decreased significantly from baseline to two months (NPRS: 7.2 ± 1.3 to 3.6 ± 1.2; VAS: 7.3 ± 2.1 to 3.3 ± 1.7; *p* < 0.0001) and functional measures showed improvements across BPFS and BCTQ assessments, accompanied by reductions in symptom burden (NPSI). Quality of life increased significantly (EQ-5D-5L index: 0.49 ± 0.23 to 0.81 ± 0.12; *p* < 0.0001), and most patients reported perceived clinical improvement. **Conclusions**: In this exploratory real-world study, Assonal^®^ supplementation was associated with clinically relevant improvements in pain intensity, functional performance, and quality of life in patients with radiculopathy and CTS, suggesting the need for further investigation of these associations in future controlled clinical studies.

## 1. Introduction

Peripheral nerve-related pain conditions, including cervical and lumbar radiculopathy and carpal tunnel syndrome (CTS), are common causes of neuropathic pain and functional limitations in clinical practice. These disorders are characterised by sensory disturbances such as burning sensations, electric shock-like pain, paraesthesia, and hyperaesthesia, which result from altered nerve signalling and peripheral nerve dysfunction [1,2,3,4]. Radiculopathy occurs due to nerve root compression or irritation, while CTS is caused by median nerve entrapment at the wrist; despite their distinct anatomical origins, both conditions share overlapping mechanisms involving neuroinflammation, altered nociceptive transmission, and maladaptive neuroplastic changes [5,6,7].

The clinical burden of these conditions extends beyond pain intensity, often affecting sleep quality, daily activities, psychological well-being, and overall quality of life [8]. Standard management strategies include nonsteroidal anti-inflammatory drugs (NSAIDs), anticonvulsants, antidepressants, rehabilitation programmes, and, in selected cases, surgical intervention [9]. However, prolonged pharmacological treatment may be limited by incomplete symptom control, adverse effects, or contraindications, particularly with NSAIDs, which are associated with gastrointestinal, renal, and cardiovascular risks when used long-term [10]. Consequently, complementary strategies aimed at modulating underlying pathophysiological mechanisms while maintaining a favourable safety profile are being explored.

In this context, nutraceutical supplementation has gained attention as an adjunct approach in managing neuropathic pain [11]. Several bioactive compounds, including B-group vitamins, magnesium, curcumin, acetyl-L-carnitine, citicoline, and palmitoylethanolamide, have been examined for their potential neuroprotective, antioxidant, and anti-inflammatory properties as they pertain to peripheral nerve dysfunction [12,13,14,15,16]. Among phytotherapeutic agents, *Gastrodia elata Blume* has attracted increasing scientific interest. Traditionally used in Asian medicine, this medicinal plant contains bioactive constituents, including gastrodin and parishin derivatives, that have shown antioxidant, anti-inflammatory, and neuromodulatory effects in experimental models [17,18,19,20].

Preclinical studies suggest that *Gastrodia elata Blume* and its active components may decrease neuronal excitability, reduce neuroinflammation, regulate oxidative stress pathways, and enhance mitochondrial function, mechanisms that could be relevant to radiculopathy, CTS, and other peripheral nerve-related pain conditions [21,22,23,24,25,26,27]. While conventional treatment remains the standard of care, interest in less invasive and supplementary strategies has increased, especially among patients seeking alternatives or complementary approaches to pharmacological therapy [22,23,24].

Despite the growing experimental and clinical interest in nutraceutical approaches targeting mechanisms involved in peripheral nerve compression disorders, real-world clinical evidence evaluating multicomponent formulations in patients with cervical or lumbar radiculopathy and CTS remains scarce. Therefore, the present study was designed as a pragmatic real-world before–after clinical investigation aimed at examining the relationship between supplementation with a novel nutraceutical formulation (Assonal^®^, Agave Group srl, Bologna, Italy), enriched with a *Gastrodia elata Blume*-based antioxidant complex (OXADIA^®^), and changes in pain intensity, functional status, symptom burden, and quality of life in adults with cervical or lumbar radiculopathy and/or CTS [28,29]. Clinical outcomes were assessed to provide a comprehensive evaluation of patient-reported improvements across pain, functional performance, and quality-of-life domains, contributing preliminary real-world clinical observations on the potential association between nutraceutical supplementation and patient-reported outcomes in these peripheral nerve compression conditions.

## 2. Materials and Methods

### 2.1. Study Design and Population

**Figure 1:**
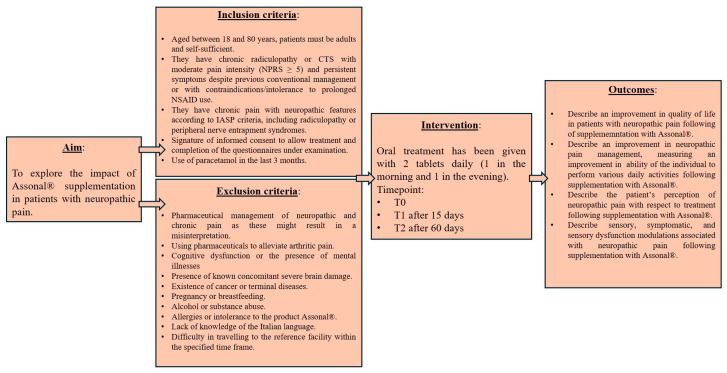
The clinical study’s summary design.

Azienda Ospedaliero Universitaria (A.O.U.) Saints (SS.) Antonio and Biagio and Cesare Arrigo Hospital in Alessandria (Italy) was the site of this single-centre, non-controlled clinical study from the time of study authorisation (Practice N° CE148/2024 of the Territorial Ethics Committee [TEC] Interagency A.O.U. Maggiore della Carità in Novara, Italy) (Figure 1). The study was performed in accordance with the Declaration of Helsinki throughout the investigation, as well as current Italian and European laws on clinical trials and the protection of personal data (Decree No. 196/2003 and 679/2016—GDPR).

As a before-and-after clinical trial, the current study used a methodological approach in which each participant was assessed at two timepoints: before the intervention (T0) and at follow-up (T1, T2). Each participant served as their own control in this approach, allowing a direct comparison of circumstances before and after the intervention [30,31]. The baseline value (T0) was defined as the mean of repeated clinical assessments collected during a 15-day pre-intervention observation period (from day −15 to day −1). During this run-in phase, patients completed the same validated questionnaires at least twice under stable clinical conditions, and the arithmetic mean of these measurements was used as the baseline value. This methodological approach was adopted to minimise the short-term symptom variability and regression-to-the-mean effects commonly observed in uncontrolled clinical studies and real-world investigations [31,32,33,34].

When it is not feasible to include an external control group for practical, logistical, or ethical reasons, the before-and-after method offers several advantages [34]. Using references from individual participants, this approach effectively reduces interindividual variability and enhances the sensitivity for detecting within-subject changes caused by the intervention. Long-term monitoring of biological and clinical markers is also possible, providing valuable insights into the durability of treatment effects over time and the temporal dynamics of response. Although the ability to draw definitive conclusions about causality is limited by the lack of a placebo or control arm, the before-and-after framework remains a reliable and instructive paradigm for examining the physiological effects of interventions in real clinical settings [35]. The present study focused on patients with peripheral nerve compression-related pain conditions, including radiculopathy and CTS, characterised by structural or functional alterations of peripheral nerve fibres. Compression of nerve roots or peripheral nerves leads to abnormal signal transduction and ectopic activity, producing pain distributed along anatomically plausible somatosensory pathways. Clinical features commonly include allodynia, sensory disturbances, hyperexcitability, and spontaneous pain, reflecting ongoing peripheral nerve dysfunction. In some patients, this neuropathic condition persists due to sustained nerve damage or incomplete recovery, resulting in continued pain despite resolution of the initial trigger or limited response to standard therapies [36]. All patients with a diagnosis of peripheral nerve compression-related pain conditions, in terms of radiculopathy and CTS, who were referred to the A.O.U. SS. Antonio, Biagio, and Cesare Arrigo of Alessandria were assessed after the study’s authorisation date to ensure they met the inclusion and exclusion criteria outlined in the following section. All participants who met the requirements were given an informed consent form. The primary investigator or a specifically designated, professionally qualified collaborator provided the supplement Assonal^®^ to patients to address any concerns and to explain all the features and evaluations included in the protocol. To proceed with the assessments in this study, an informational letter was written to the treating physician for each patient who completed the informed consent form. Although the enrolled patients presented with different clinical diagnoses (lumbar or cervical radiculopathy and CTS), both conditions involve peripheral nerve dysfunction associated with neuropathic features and pain-related functional impairment. For the purpose of this exploratory real-world study, the population was analysed as a single cohort characterised by peripheral nerve compression-related pain, while acknowledging the distinct anatomical and pathophysiological substrates of each condition. Outcome measures were selected to capture shared symptom dimensions (pain intensity, sensory descriptors, and quality of life) as well as condition-specific functional domains, without implying pathophysiological equivalence between diagnoses. This approach reflects routine clinical practice, where management strategies are frequently symptom-oriented and function-driven. No formal comparative analyses between diagnostic subgroups were planned or performed, as the study was neither powered nor designed to detect disease-specific differences.

The study was divided into four phases according to different evaluation timepoints:

- T0 (enrolment of the subject in the study after monitoring phase from day –15 to day –1): following the initial submission of the questionnaires and the explanation of the treatment administration dose (two Assonal^®^ tablets daily), this group was designated as the control group for the subsequent phases (Before).
- T1: After 15 days of oral treatment (two tablets a day of Assonal^®^), evaluations with questionnaire administration followed.
- T2: After two months of treatment taken orally (two tablets per day of Assonal^®^), final evaluations were conducted with the administration of the questionnaires.

Each patient was scheduled for a visit at the A.O.U at each assessment time point. SS. Antonio, Biagio, and Cesare Arrigo of Alessandria were the locations of assessments administered using questionnaires with the attending physician’s approval.

### 2.2. Eligibility Criteria of the Study Population

#### 2.2.1. Inclusion Criteria

- Patients were required to be aged between 18 and 80 years and needed to be self-sufficient.
- Eligible participants had chronic radiculopathy or CTS with moderate pain intensity (NPRS ≥ 5) and persistent symptoms despite previous conventional management or with contraindications/intolerance to prolonged NSAID use.
- They had chronic pain with neuropathic features according to IASP criteria, including radiculopathy or peripheral nerve entrapment syndromes.
- The patient was required to sign their informed consent prior to treatment and complete the questionnaires under examination.
- Prior use of paracetamol within the 3 months preceding enrolment was permitted and did not affect eligibility.

#### 2.2.2. Exclusion Criteria

- Pharmaceutical management of neuropathic and chronic pain (such as acetyl-L-carnitine, tricyclic antidepressants, opioids, and antiepileptics), as these might result in a misinterpretation.
- Using pharmaceuticals to alleviate arthritic pain would lead to inaccurate data interpretation.
- Cognitive dysfunction or the presence of mental illnesses, as individuals with these conditions may fill out the questionnaires for the final evaluation incorrectly.
- The presence of known concomitant severe brain damage.
- The existence of cancer or terminal diseases, as patients with cancers may be prescribed certain treatments that might obstruct the study’s therapy.
- Pregnancy or breastfeeding.
- Alcohol or substance abuse.
- Allergies or intolerance to the product Assonal^®^, as this would cause serious adverse side effects.
- Lack of knowledge of the Italian language, as it would not be possible to complete the necessary questionnaires.
- Difficulty travelling to the reference facility within the specified time frame.

According to the study’s operational definition, a verbal rating scale (VRS) score of “Moderate” or higher or a numerical pain rating scale (NPRS) score of ≥5 indicated that a participant experienced at least moderate pain [37,38]. Since NPRS/VRS or Visual Analogue Scale (VAS) scores > 5 were used as inclusion criteria in earlier clinical studies, this threshold aligns with conventional clinical practice. It helps optimise the chance of detecting meaningful changes while reducing potential floor effects [39].

All additional nutritional supplements, herbal extracts, and natural products with known or suspected analgesic, anti-inflammatory, or neuromodulatory properties were to be avoided by patients during the research period. This guideline was followed throughout the screening process and at every follow-up appointment. Participants were instructed to maintain stable background therapies throughout the study period. The initiation of new analgesic, anti-inflammatory, or neuromodulatory treatments during follow-up was considered a protocol deviation and was documented accordingly. This approach aligns with recommendations for pragmatic clinical investigations that aim to reduce confounding factors while preserving real-world applicability [31,34]. Rescue medication with paracetamol was permitted if required, and its use was recorded at each follow-up visit to monitor potential confounding effects.

### 2.3. Product Under Investigation

Assonal^®^ is a multicomponent nutraceutical formulation containing bioactive compounds with reported antioxidant, metabolic-supportive, and neuroprotective properties, as described in the experimental and clinical literature [40]. The full quantitative composition per daily dose (2 tablets) is reported in Table 1.

The formulation includes a standardised *Gastrodia elata Blume*-derived antioxidant complex (OXADIA^®^), providing a characterised source of phytochemical compounds investigated for their potential modulation of oxidative stress and neuroinflammatory pathways [41,42], and replaces a component present in the original formulation, α-lipoic acid (ALA) [20]. The OXADIA^®^ extract is defined by a specific phytochemical composition (% w/w dry extract), including polysaccharides (10.9 ± 0.3%), polyphenols (3.2 ± 0.1%, including vanillic acid and gastrodin derivatives), triterpenoids (2.0 ± 0.37% *w*/*w* dry extract), and lignans (0.05 ± 0.002% *w*/*w* dry extract), as determined by UV–Vis spectrophotometry (Infinite 200 Pro MPlex plate reader, Tecan, Männedorf, Switzerland) and High-Performance Liquid Chromatography (HPLC) analyses (Agilent, Santa Clara, CA, USA).

Assonal^®^ also contains acetyl L-carnitine (ALC), which is naturally present in tissues and has a biological role in mitochondrial energy metabolism and neuronal cellular homeostasis [43]. Its best-known function is to produce energy through the process of β-oxidation of fatty acids, supporting the activity of mitochondria [44]; citicoline, an endogenous molecule essential during the synthesis of membrane phospholipids [45]; and B vitamins (B1, B2, B5, B6, and B12), which contribute to normal energy metabolism and protection from oxidative stress [46,47].

Enrolled patients were instructed to take two tablets daily (one in the morning and one in the evening) with water, according to the manufacturer’s recommendations. Treatment adherence and dosing schedule were standardised across participants. The administration regimen was identical for all patients, regardless of pain type or anatomical location, with no dose modifications during the study. No body weight-adjusted dosing (mg/kg) was applied, as the formulation was administered as a standardised nutraceutical intended for adult use.

### 2.4. Endpoints

Patients with concomitant CTS were analysed as an exploratory subgroup, using condition-specific outcome measures (Boston Carpal Tunnel Questionnaire—BCTQ), to ensure that we described symptom and functional trends over time rather than drawing disease-specific efficacy conclusions. These analyses aimed to be descriptive and hypothesis-generating rather than confirmatory.

#### 2.4.1. Primary Endpoint

The primary endpoint of the study was the reduction in perceived pain intensity, assessed using both the NPRS and the VAS at predefined timepoints T0, T1, and T2. The NPRS (also called the Numeric Rating Scale—NRS) is a unidimensional 11-point scale widely used to evaluate pain intensity in adults, including those with chronic pain related to rheumatic or neuropathic disorders [48,49]. It consists of a linear scale from 0 (“no pain”) to 10 (“worst imaginable pain”), allowing patients to easily quantify their pain perception. The tool can be administered verbally or graphically, including by telephone, and usually takes less than 1 min to complete. Unlike the VAS, the NPRS does not require fine visual–motor coordination, making it suitable for a wider range of patients. Additionally, the scale has shown strong test–retest reliability, particularly among literate subjects (r = 0.96) compared to illiterate participants (r = 0.95). Its main advantages include simplicity, reproducibility, high sensitivity to small changes in pain intensity, and excellent cross-cultural applicability [50]. The VAS, on the other hand, is a continuous measurement tool comprising a 10 cm horizontal line anchored by the descriptors “no pain” and “worst pain imaginable.” Patients are asked to mark the position on the line that best reflects their current pain level, and the score is derived by measuring the distance in millimetres from the “no pain” anchor [51]. Compared to the NPRS, the VAS may detect more subtle variations in pain perception. However, it can be somewhat more challenging to complete, particularly for older adults or those with limited manual dexterity. Nevertheless, it remains a validated, reliable, and sensitive instrument for assessing pain intensity in both clinical practice and research [52].

#### 2.4.2. Secondary Endpoint

The secondary endpoints were:

- Quality of life was measured using the EuroQol 5-Dimension 5-Level questionnaire (EQ-5D-5L), a standardised and validated tool for assessing general health status [53,54]. The instrument consists of two sections: a descriptive system and a VAS. The descriptive part evaluates five key dimensions—mobility, self-care, usual activities, pain/discomfort, and anxiety/depression—each scored from 1 (no problems) to 5 (extreme problems or inability to perform the activity). The VAS part records the participant’s self-rated health on a vertical scale from 0 to 100, where 0 indicates the worst imaginable health state and 100 the best possible health, in response to the question, “How good or bad is your health today?” Combining the scores from both sections, the EQ-5D-5L offers a comprehensive assessment of overall quality of life, capturing both functional and subjective aspects of well-being.
- Changes in pain intensity, functional impairment, and patient-reported symptom burden were evaluated using validated clinical instruments commonly used in peripheral nerve compression conditions [52,55,56], specifically the Back Pain Functional Scale (BPFS) for lumbar and cervical pain and BCTQ for CTS [57,58] at T0, T1, and T2. The BPFS comprises 12 items assessing performance across domains, including work activities, hobbies, household chores, bending, putting on shoes or socks, lifting, sleeping, standing, walking, climbing stairs, sitting, and driving. The scale is grounded in the International Classification of Functioning, Disability and Health (ICF) framework proposed by the World Health Organisation (WHO) and has a maximum score of 60 points. The highest score indicates the patient’s greatest level of physical independence and functional capacity during the acute or subacute phase of back pain. Each item is rated on a 5-point Likert scale (1 = no difficulty performing the activity; 2 = little difficulty; 3 = moderate difficulty; 4 = great difficulty; 5 = unable to perform the activity) [59]. For consistency with standard interpretation, item scores were transformed so that higher total scores reflected better functional status, yielding a total BPFS score ranging from 0 (complete functional limitation) to 60 (full functional capacity). This scoring approach allows direct interpretation of changes over time, with higher values indicating functional improvement.
- The BCTQ, also known as the Levine Questionnaire, is a self-assessment tool specifically designed to measure symptom severity and functional disability in individuals with CTS [55]. The scale comprises two subsections: the Symptom Severity Scale (SSS), consisting of 11 items that evaluate the intensity and frequency of core symptoms (pain, paraesthesia, numbness, weakness, sleep disturbances), and the Functional Status Scale (FSS), made up of 8 items that assess the level of difficulty in performing daily activities (such as writing, combing hair, opening a jar, or holding a book). Each item is rated on a 5-point Likert scale, with higher scores indicating more severe symptoms or reduced hand function. The average scores of the two subscales can be analysed separately, offering a sensitive, quantitative measure of clinical change over time. A validated Italian version exists, with psychometric properties comparable to the original, making the BCTQ a key tool for assessing patients with CTS [56].
- The evaluation of patients’ perceived improvement following treatment was conducted using the Global Perceived Effect (GPE) scale, a 7-point Likert-type questionnaire designed to capture the individual’s subjective impression of change [60]. Through this measure, each participant reports how their condition has evolved compared with the pre-treatment phase, selecting one of seven possible responses: 1 = very much worse, 2 = moderately worse, 3 = slightly worse, 4 = no change, 5 = slightly improved, 6 = moderately improved, and 7 = much improved. The GPE is widely used in both clinical practice and research as a straightforward and dependable indicator of treatment-related improvement, particularly within musculoskeletal, neurological, and rehabilitation contexts.
- The modulation of sensory symptoms and the overall severity of neuropathic pain dysfunction following Assonal^®^ supplementation were assessed using the Neuropathic Pain Symptom Inventory (NPSI). The NPSI is a validated questionnaire that is specifically designed to quantify and characterise the different dimensions of neuropathic pain, enabling a detailed assessment of both intensity and qualitative aspects of symptoms [61]. It evaluates average pain intensity and distinguishes between various pain descriptors, such as burning, squeezing, electric shock-like sensations, pressure, tingling, and evoked pain, thereby providing a multidimensional profile of the patient’s sensory experience. Each item is scored on an 11-point numeric scale, where 0 corresponds to “no pain” and 10 to “the worst imaginable pain”. This scale has been validated in several languages, including Italian [62], and has demonstrated strong psychometric properties, including sensitivity to change over time and the ability to discriminate between different neuropathic pain syndromes. As such, the NPSI represents a reliable and comprehensive tool for evaluating changes in sensory modulation and symptom burden in patients with neuropathic pain conditions [63].

### 2.5. Statistical Analysis

Sample size estimation was carried out using G*Power 3 software, focusing on the primary outcome of pain intensity. Assuming a mean within-subject reduction in pain score of 2.4 points after supplementation with Assonal^®^, consistent with clinically meaningful reductions in musculoskeletal pain reported in the literature [64], a minimum sample size of 36 participants was calculated to achieve 80% power at a two-sided α level of 0.05. To accommodate a potential dropout rate of approximately 25%, initial enrolment was increased accordingly. Statistical analyses were conducted using GraphPad Prism (version 10.2.3; GraphPad Software, San Diego, CA, USA). Continuous variables are expressed as mean ± standard deviation (SD). Data normality was assessed with the Shapiro–Wilk test. Longitudinal changes in pain intensity, neuropathic symptom burden, functional outcomes, and quality-of-life measures throughout the supplementation period (T0, T1, and T2) were analysed using repeated-measures statistical methods. For variables with a normal distribution, repeated-measures ANOVA with the Geisser–Greenhouse correction was applied to address potential violations of sphericity. When data did not meet normality assumptions, the Friedman test was used. Post hoc pairwise comparisons were performed using Tukey’s or Dunn’s multiple comparison tests, as appropriate, with adjustments for multiple testing. All statistical tests were two-sided, and *p*-values < 0.05 were deemed statistically significant. Given the exploratory nature of the study and the absence of a comparator group, statistical analyses were primarily intended to quantify within-subject longitudinal changes rather than to demonstrate causal treatment effects, in line with methodological recommendations for real-world and before–after clinical investigations. Effect sizes (where applicable) were interpreted descriptively to emphasise clinical relevance alongside statistical significance. No interim analyses were planned or conducted. Subgroup analyses by pain location (cervical radiculopathy, lumbar radiculopathy and CTS) status were performed in an exploratory manner. These analyses, reported in Appendix A, were descriptive and not powered for formal inferential comparisons between subgroups.

## 3. Results

After the screening phase, 78 patients were assessed for eligibility. Thirty-six patients met the inclusion criteria and were enrolled, while 42 were excluded based on predefined criteria, including concomitant pharmacological treatments that could interfere with pain assessment, cognitive or psychiatric conditions affecting the reliability of the questionnaire, serious neurological or systemic diseases, pregnancy or breastfeeding, known intolerance to the study product, insufficient knowledge of the Italian language, or logistical limitations. Patient selection and reporting followed recommendations for observational before–after clinical studies, and participant flow is summarised in the study diagram.

**Figure 2:**
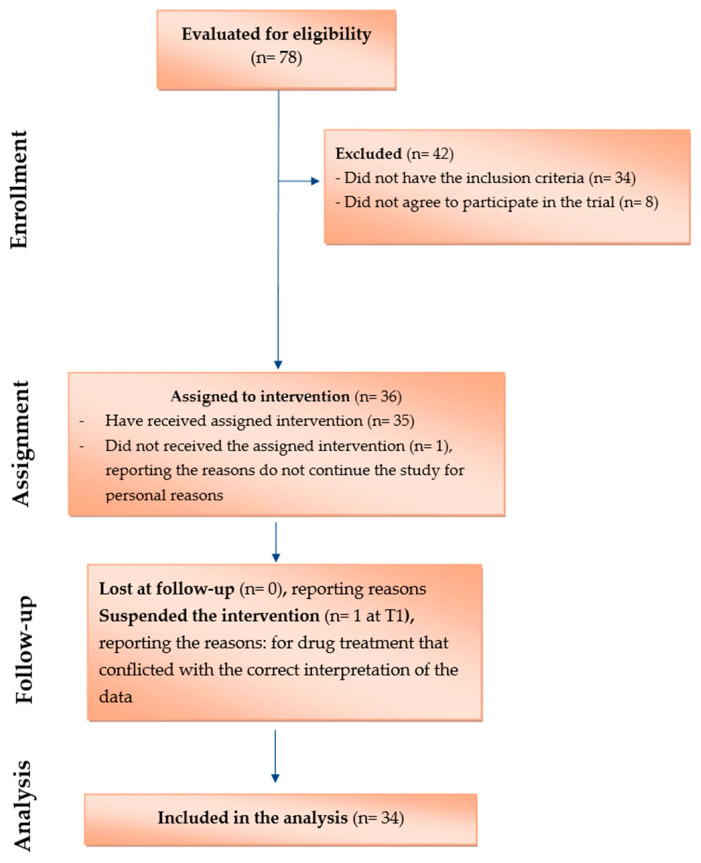
The flow chart illustrates the various steps of the before-and-after clinical research.

The trial began with 20 male and 16 female patients suffering from persistent pain related to cervical or lumbar radiculopathy and/or CTS lasting more than three months (Table 2). During the 15-day monitoring period (from T −15 to T −1) before the start of the operational phase (T0) and the administration of Assonal^®^, one male patient at T0 stated that he no longer wished to participate in the clinical study for personal reasons. Additionally, one female patient withdrew from the trial at T1 due to starting therapeutic practices that conflicted with the correct interpretation of the experimental results (Figure 2), corresponding to a low dropout rate (5.6%). Given the minimal attrition, no sensitivity analyses were performed, and a post hoc power analysis was deemed uninformative, as it did not add inferential value beyond effect estimates and confidence intervals. As a result, 34 patients completed the clinical trial. Of these, 41% had cervical radiculopathy at C5–C6, and 59% experienced lumbar pain related to L4–L5 radiculopathy. Furthermore, CTS was present in 44% of the 34 patients. All participants followed the same fixed dosing regimen across the study period. Concomitant treatments were kept stable throughout the study period. Patients receiving neuropathic-specific pharmacological therapies (see the exclusion criteria) were excluded at baseline, and no initiation or modification of long-term analgesic or non-pharmacological treatments was allowed during follow-up. Paracetamol was permitted as rescue medication and was available to all patients. Its use was on an as-needed basis and was infrequent and irregular. Detailed data are reported in Appendix A (Table A1).

Throughout the cohort, consistent improvements were observed in pain intensity, functional outcomes, and quality-of-life measures, with 32 out of 34 participants reporting perceived clinical improvement during the supplementation period, while two reported no improvement or deterioration. The majority of enrolled patients reported a clinically relevant improvement in functional outcomes and pain intensity during the supplementation period, as evidenced by consistent reductions across multiple validated neuropathic pain scales. In detail, 94.1% of patients achieved a ≥30% reduction in NPRS, indicating a clinically meaningful responder rate. Adverse events were generally mild and transient. No serious adverse events were observed, and no participant discontinued the intervention due to adverse effects (see Table A2, Appendix A).

### 3.1. Assonal^®^’s Impact on Perceived Pain Intensity

**Figure 3:**
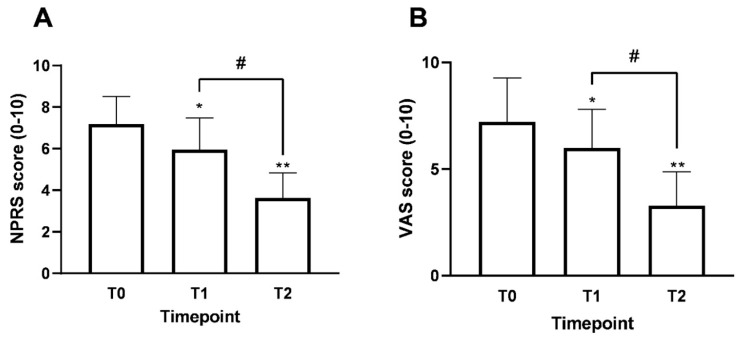
Results for the study’s primary endpoint (pain intensity) before and after taking Assonal^®^. The findings for the NPRS score and VAS are shown in (**A**) and (**B**), respectively. Data are presented as the mean ± SD of self-reported scores obtained after administering the questionnaire to 34 patients at each timepoint. In (**A**) * *p* = 0.002 T0 vs. T1; ** *p* < 0.0001 T0 vs. T2; # *p* < 0.0001 T1 vs. T2. In (**B**) * *p* < 0.05 T0 vs. T1; ** *p* < 0.0001 T0 vs. T2; # *p* < 0.0001 T1 vs. T2.

The analysis of pain progression during treatment, the main endpoint of the clinical study, was carried out using two extensively validated tools for pain evaluation: NPRS and VAS (Figure 3). The combined application of both scales enabled us to obtain a clearer, more reliable picture of the patient’s pain intensity over time. The results reveal an overlapping trend between the two measurements, confirming the data’s internal consistency and suggesting a consistent pattern of patient-reported changes over time.

The NPRS (Figure 3A) baseline value (T0) was 7.2 ± 1.3. After two weeks of treatment with Assonal^®^ (T1), the pain intensity decreased significantly to 5.9 ± 1.5 (*p* = 0.002). This early reduction corresponds to an initial improvement in reported pain. Compared to T0, the decrease was 25%. After 2 months of supplementation (T2), the NPRS score reached 3.6 ± 1.2, indicating a 50% decrease from baseline and a 39% decrease from T1. This improvement was highly statistically significant (*p* < 0.0001 compared to T0 and T1). The extent of the reduction reflects the stabilisation of the observed patient-reported improvements.

A similar pattern was confirmed by the VAS scores (Figure 3B). The baseline (T0) value was 7.3 ± 2.1. During treatment, the scores showed a consistent decline, reaching a mean of 3.3 ± 1.7 at the final recorded timepoint (T2), which represents a 55% reduction from the initial value. The differences between T0 and T2 were statistically significant (*p* < 0.0001), further indicating a consistent reduction in patient-reported pain over time.

Overall, the data collected through NPRS and VAS demonstrate a clear and consistent decrease in pain intensity across the various phases of treatment with Assonal^®^.

A subgroup analysis stratified by pain location (cervical radiculopathy, lumbar radiculopathy, and CTS) showed a consistent reduction in pain intensity over time across all groups. The observed trends appeared comparable across subgroups, although formal inferential comparisons were underpowered in this exploratory analysis (see Appendix A, Table A3 and Table A4).

### 3.2. Analysis of Secondary Endpoints: Patient Perception at Treatment, Symptom Modulation and Quality-of-Life Improvements

The patient’s subjective assessment of clinical improvement was measured using the GPE, a widely used method for quickly and easily quantifying symptom progression from the patient’s perspective. The collected scores showed a positive trend, suggesting an increase in patient-reported satisfaction during the supplementation period.

**Figure 4:**
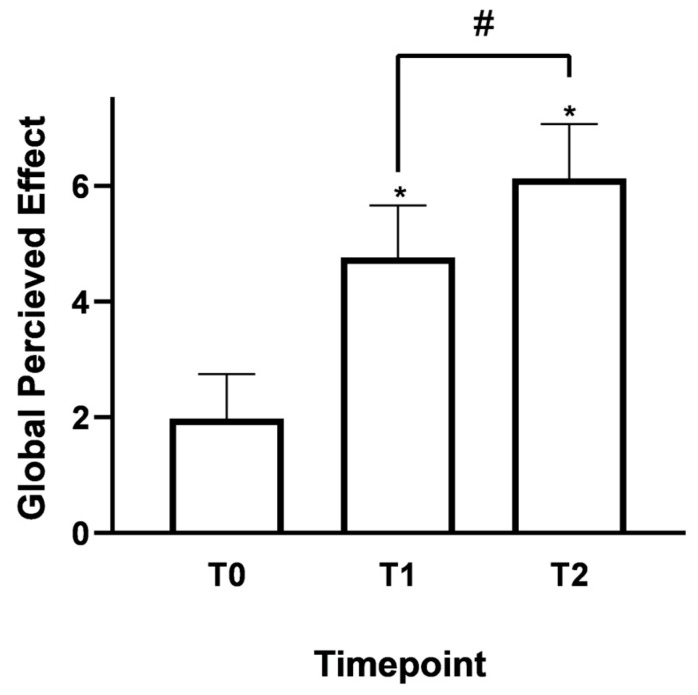
Data on treatment satisfaction (GPE score) were collected between T0 and T2 in accordance with the established evaluation protocol. The data are presented as the mean ± SD of self-reported scores obtained after administering the questionnaire to 34 patients at all examination timepoints. * *p* < 0.0001 T0 vs. T1 and T2; # *p* < 0.05 T1 vs. T2.

As shown in Figure 4, after starting Assonal^®^, the mean GPE score was 4.8 ± 0.9 at the beginning of the two-week supplementation period (T1), indicating significant improvement in pain-related and functional symptoms. This increase, which was evident soon after starting therapy, aligns with reductions in pain intensity reported by the NPRS and VASs. At T1, the mean GPE score was 4.8 ± 0.9, indicating early perceived improvement (*p* < 0.0001 vs. T0). At T2, the score increased further to 6.1 ± 0.9 (*p* < 0.05 vs. T1 and *p* < 0.0001 vs. T0), reflecting a shift toward “moderately” to “much improved” categories. These results suggest patient-reported improvements in overall condition and satisfaction during the supplementation period, without implying a causal effect of the intervention.

**Figure 5:**
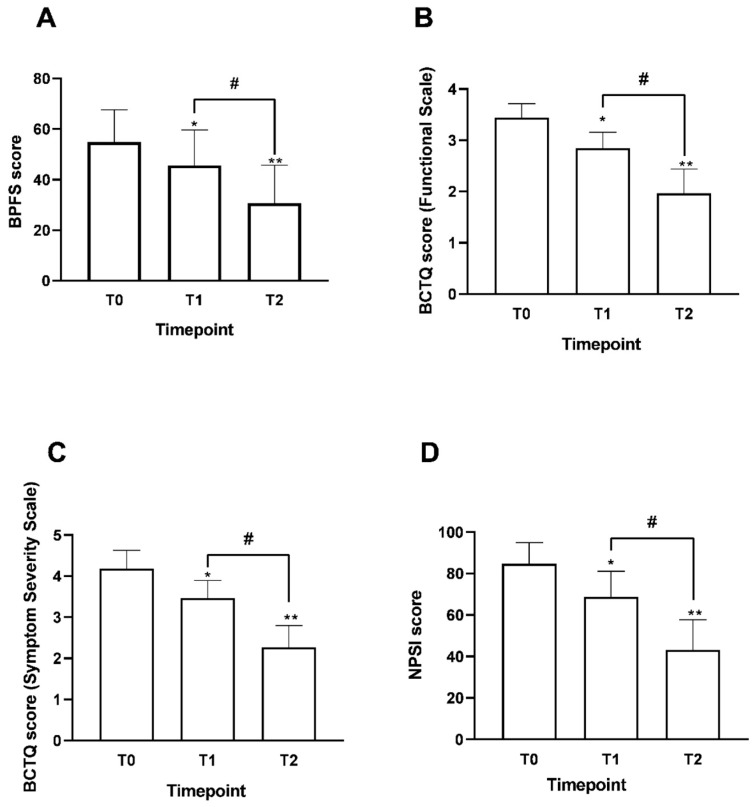
Findings on improvements in pain conditions and symptom modulation from T0 to T2 following Assonal^®^ oral intake, in accordance with the study’s examination protocol. In (**A**), the findings related to the BFPS are shown; in (**B**), the results concerning BCTQ (Functional scale) are presented; in (**C**), the data related to the BCTQ (Symptom Severity Scale) are displayed; and in (**D**), the results of the NPSI score are shown. Data represent the mean ± SD of self-reported scores collected from 34 patients across all stages of the examination protocol. For BCTQ data (**B**,**C**), they refer to the assessments of the 15 patients also affected by CTS. In (**A**) * *p* < 0.05 T0 vs. T1; ** *p* < 0.0001 T0 vs. T2; # *p* = 0.0002 T1 vs. T2. In (**B**) * *p* = 0.0002 T0 vs. T1; ** *p* < 0.0001 T0 vs. T2; # *p* < 0.0001 T1 vs. T2. In (**C**) * *p* = 0.0004 T0 vs. T1; ** *p* < 0.0001 T0 vs. T2; # *p* < 0.0001 T1 vs. T2. In (**D**) * *p* < 0.0001 T0 vs. T1; ** *p* < 0.0001 T0 vs. T2; # *p* < 0.0001 T1 vs. T2.

Using the combined assessment of the BFPS and BCTQ scales, the study observed improvements in functional performance, while neuropathic sensory symptom burden was evaluated specifically with the NPSI (Figure 5A). These findings indicate that the supplementation treatment yielded a gradual, clinically meaningful response over the observed period.

The BFPS baseline value (T0) was 54.9 ± 12.3, indicating a relevant functional limitation. After supplementation, scores progressively increased. At T1, BFPS reached 45.7 ± 13.6 (*p* < 0.05 vs. T0), and further improved at T2 to 30.7 ± 14.7 (*p* < 0.0001 vs. T0), reflecting a substantial recovery of daily functional performance. These modulations suggest an increase in patients’ ability to carry out daily activities, with fewer symptom-related limitations, as shown by the progressive decline in BFPS scores.

In line with the previous description, significant data were also collected within the CTS context. The BCTQ Functional Scale (Figure 5B) assessed the extent of symptom-related functional impairment, with an initial mean score of approximately 3.4 ± 0.3 at T0, indicating a substantial limitation in daily activities, evenly distributed among CTS patients. At two weeks (T1) of Assonal^®^ intake, a significant reduction in the functional score was observed, with an average of approximately 2.8 ± 0.4 (a 20% reduction compared to T0; *p* = 0.0002). This improvement was even more evident at T2, where the score decreased to approximately 1.9 ± 0.6, indicating greater statistical significance relative to T0 (44% reduction; *p* < 0.0001) and a significant improvement compared to T1 (32% reduction; *p* < 0.0001). Overall, the progressive decrease in BCTQ values suggest a notable functional recovery over time, positively affecting patients’ ability to perform daily activities. Meanwhile, the BCTQ Symptom Severity Scale (Figure 5C) allowed us to evaluate the severity of symptoms reported by patients. At baseline (T0), the mean score was approximately 4.2 ± 0.5, indicating marked symptomatology. At T1, patients reported a significant reduction in score to 3.5 ± 0.4 (18%; *p* = 0.0004). The improvement continued at T2, where the value dropped to roughly 2.3 ± 0.5, indicating further modulation than at T0 (45% reduction; *p* < 0.0001) and continued improvement compared to T1 (34% reduction; *p* < 0.0001).

Parallel improvements were observed in neuropathic symptom burden, as indicated by the NPSI score (Figure 5D). At baseline (T0), participants had a mean NPSI score of 84.7 ± 10.2, signifying a clinically relevant burden of radicular and compression-related sensory symptoms. This elevated baseline value indicates the high sensory symptom burden of the enrolled cohort, composed of patients with persistent moderate-to-severe radiculopathy or CTS referred for clinical management after previous therapeutic attempts. It aligns with enrolment criteria that specify moderate-to-severe symptom intensity.

A notable reduction occurred after just two weeks of treatment, with the score dropping to 68.6 ± 12.2 at T1 (a 24% reduction), which was highly statistically significant (*p* < 0.0001 vs. T0). This positive progression became even more evident at T2, when the mean NPSI decreased further to 43.3 ± 14.5, representing a 49% reduction from baseline and indicating a clinically relevant change over time (*p* < 0.0001 vs. T0 and T1).

Subgroup analyses by pain location (cervical radiculopathy, lumbar radiculopathy with and without CTS) showed a progressive decrease in NPSI scores across all groups over time. The patterns of improvement were broadly similar across subgroups; however, this exploratory analysis was not sufficiently powered to support formal between-group comparisons (see Appendix A, Table A5).

**Figure 6:**
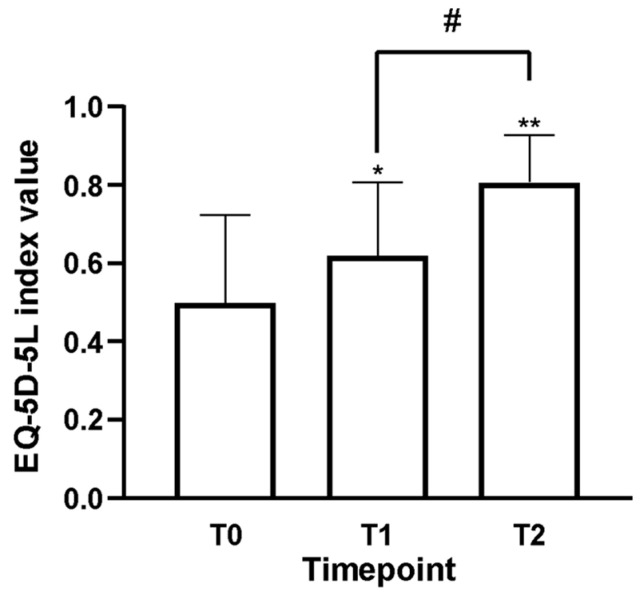
Findings on the improvement in quality of life observed in enrolled patients before and after Assonal^®^ intake. The EQ-5D-5L questionnaire data are presented. Data are shown as the mean ± SD of the self-reported scores collected from 34 patients across all stages of the examination protocol. * *p* < 0.05 T0 vs. T1; ** *p* < 0.0001 T0 vs. T2; # *p* = 0.0004 T1 vs. T2.

Furthermore, using the EQ-5D-5L (Figure 6) enabled us to accurately monitor changes in patients’ reported quality of life during the assessment period. At T0, the 34 patients had a mean score of 0.49 ± 0.23, indicating significant discomfort and reduced quality of life due to persistent neuropathic pain. This finding aligns with the original clinical presentation, which included ongoing symptoms and functional limitations caused by chronic pain. Following the start of oral supplementation with Assonal^®^, a notable improvement was observed after just 2 weeks (T1), with a score increase to 0.62 ± 0.19 (*p* < 0.05 vs. T0). This represents a 21% improvement in quality of life compared to T0. The positive trend became even clearer at T2, when the EQ-5D-5L score reached 0.81 ± 0.12, demonstrating significant statistical differences compared to T0 (*p* < 0.0001) and T1 (*p* = 0.0004). Overall, the quality of life increased by 38% from baseline and by 24% from the first follow-up.

Subgroup analyses by pain location (cervical radiculopathy and lumbar radiculopathy, with and without CTS) showed a consistent upward trend in EQ-5D-5L scores across all groups over time. Improvements appeared similar between subgroups; however, this exploratory analysis lacked sufficient statistical power to support formal comparisons between groups (see Appendix A, Table A6).

## 4. Discussion

Peripheral nerve compression-related pain conditions, including cervical and lumbar radiculopathy and CTS, represent a significant clinical challenge, frequently impairing daily activities, physical functioning, and overall quality of life [65]. Conditions such as low back pain, cervical radiculopathy, and CTS are very common and are frequently linked with ongoing pain, functional limitations, and reduced well-being [66]. Despite the availability of traditional pharmacological treatments, many patients still experience notable symptoms, emphasising the need for complementary or additional strategies [67]. Recently, nutraceutical interventions have attracted attention for their potential to influence neuroprotection, pain perception, and neural function, as well as for a promising safety profile. These features suggest that nutraceuticals could be a valuable supplement to standard treatments, helping to reduce pain, support functional recovery, and enhance quality of life [68].

A potential limitation of this study is the inclusion of patients with different clinical diagnoses, specifically lumbar or cervical radiculopathy and CTS. However, this heterogeneity was deliberate and reflects real-world clinical situations in which peripheral nerve compression-related pain conditions share a common pathophysiological basis across anatomical conditions. All participants met the IASP criteria for neuropathic pain and showed similar symptom profiles, including sensory disturbances and functional impairments. As a result, the study aimed to explore the relationship between supplementation and improvements in neuropathic pain features rather than to evaluate disease-specific efficacy. The consistent improvements across pain intensity, neuropathic symptom burden, functional scales, and quality-of-life measures suggest that the observed modulations may be consistent with modulation of mechanisms commonly involved in peripheral nerve compression-related pain.

One particularly relevant element in the pathophysiology of pain is the contribution of *Gastrodiae elata*, a medicinal plant extensively used in traditional Asian medicine that is gaining increasing recognition in modern biomedical research for its neuroprotective, antioxidant, and anti-inflammatory activities [41]. Its main bioactive constituents, notably gastrodin and parishin derivatives, have been shown to modulate multiple signalling pathways implicated in neuroinflammation. These compounds reduce microglial activation, lower the production of pro-inflammatory cytokines such as tumour necrosis factor α (TNF-α) and interleukin-1β (IL-1β), and suppress excessive reactive oxygen species (ROS) generation [69,70]. Furthermore, *Gastrodiae elata* has been shown to maintain mitochondrial homeostasis by stabilising the membrane potential, reducing oxidative stress, and supporting cellular energy metabolism [71]. Importantly, experimental evidence also indicates a protective effect on myelin integrity, aiding the maintenance of axonal conduction and structural nerve stability. By mitigating glutamate-mediated excitotoxicity and supporting both mitochondrial function and myelin preservation, *Gastrodiae elata* exerts a multifaceted neuroprotective influence [72,73]. From a translational perspective, these mechanisms provide a biologically plausible rationale for potential benefits in peripheral nerve compression-related pain conditions, thanks to *Gastrodiae elata*’s ability to intervene in peripheral and central sensitisation mechanisms, to improve axonal transmission, and to preserve neuronal function [70]. The inclusion of *Gastrodiae elata* (OXADIA^®^) in the Assonal^®^ formulation allows the combination of molecules with proven neuroprotective effects (e.g., ALC, vitamins B) with a phytocompound that enhances the overall effect, particularly in reducing oxidative stress and neuroinflammation, two key biological processes in peripheral nerve compression-related pain conditions.

Indeed, in our trial, both NPRS and VAS scores showed a consistent and progressive decrease: from baseline values of 7.2 ± 1.3 and 7.3 ± 2.1, respectively, down to 3.6 ± 1.2 (NPRS) and 3.3 ± 1.7 (VAS) after two months, corresponding to reductions of approximately 50–55%. These reductions surpass the minimum clinically important difference (MCID) for both scales (~1.5–2.5 points), confirming that Assonal^®^ supplementation was associated with statistically significant and clinically meaningful reductions in pain intensity [74,75]. This is further supported by responder analysis, with 94.1% of patients achieving a ≥30% reduction in NPRS, indicating a high proportion of clinically meaningful responders. Exploratory subgroup analyses appear to support a consistent pattern of improvement across different pain locations and CTS status. Although these findings should be interpreted with caution, given the exploratory and underpowered nature of the comparisons, they may suggest a broadly consistent pattern of change across subgroups. Consequently, these results are hypothesis-generating and warrant confirmation in larger, adequately powered studies.

In the context of chronic pain, clinical trials have focused on incorporating the active ingredient *Gastrodiae elata* into standard migraine therapy, improving pain control, decreasing attack frequency and duration, and improving quality of life compared to conventional therapy alone [76,77]. Although much of the clinical evidence for *Gastrodiae elata* originates from migraine studies, a growing body of preclinical research supports its potential benefits in models of neuropathic and inflammatory pain. In rodent models of chemotherapy-induced peripheral nerve compression-related pain, *Gastrodiae elata* extracts alleviated mechanical allodynia and thermal hyperalgesia, reduced pro-inflammatory cytokines (IL-6, TNF-α, IL-1β) in the sciatic nerve, spinal cord, and dorsal root ganglia (DRG), and upregulated neuroprotective pathways, including sirtuin-1 (SIRT1). These effects have been observed in 3D in vitro models and involve modulation of NF-κB, MAPK pathways, and GABAergic activity, which may help counteract neuronal hyperexcitability [20,25,77,78]. These findings suggest modulation of neuroinflammation, oxidative stress, and neuronal survival pathways. Since these pathophysiological mechanisms largely overlap with those underlying chronic and neuropathic musculoskeletal pain, the neuroprotective and anti-inflammatory actions of *Gastrodiae elata* provide a biologically plausible rationale for its inclusion in Assonal^®^ and support the hypothesis on its potential relevance in a broader spectrum of pain conditions beyond migraine.

The observed functional and symptomatic improvements after Assonal^®^ supplementation may have a mechanistic basis in the multimodal pharmacology of its constituents, particularly *Gastrodiae elata* and the well-documented agents, ALC and B vitamins [79,80,81]. Preclinical data indicate that *Gastrodiae elata*, related extracts, and other substances such as ALC and vitamins B, citicoline, attenuate neuroinflammation, oxidative stress, and nociceptive hyperexcitability in models of peripheral nerve injury and neuropathic pain, suppressing pro-inflammatory cytokines and modulating neuroprotective pathways (e.g., Nrf2 redox signalling, suppression of inflammasome activation, and regulation of neurotrophic factors). These effects can translate into reduced pain signalling and improved nerve recovery over time [82,83,84]. Beyond preclinical data, emerging clinical evidence supports the use of nutraceutical combinations for musculoskeletal and neuropathic pain. 

Taken together, these findings provide preliminary clinical observations suggesting that multicomponent nutraceutical formulations, such as Assonal^®^, may be associated with improvements in pain-related symptoms, functional performance, and quality of life in conditions associated with peripheral nerve compression. The notable results, including reductions in pain scores, consistent improvements in functional scales (BFPS for low back/cervical pain; BCTQ for CTS), and a decrease in neuropathic symptom burden (NPSI), align with mechanisms described in preclinical research. This mechanistic consistency may align with the patient-reported changes observed during the supplementation period. Using the EQ-5D-5L, we reliably tracked changes in quality of life. The positive trend up to T2 indicates a strong enhancement in patients’ well-being throughout the supplementation period, alongside high patient satisfaction (GPE score). The 21% improvement from T1 to T2 may reflect ongoing changes in patients’ perceived condition and satisfaction with the intervention. In stratified integrative analyses, consistent patterns of change, similar to those observed for VAS and NPRS, were also identified in secondary outcomes (NPSI and EQ-5D-5L), as reported in Appendix A. Exploratory subgroup analyses further suggested a broadly consistent pattern of changes across different pain locations and CTS statuses. Although these findings should be interpreted with caution due to the exploratory nature and limited statistical power of these comparisons, they may indicate a generally homogeneous response across subgroups.

A further methodological consideration relates to the dosing regimen. All participants received a fixed dosage of two tablets per day, without body weight adjustment (mg/kg) or modifications based on pain type or anatomical location. This approach was chosen to reflect the standardised use of the nutraceutical in routine clinical practice and to ensure uniformity of administration across the study population. While weight-adjusted dosing could theoretically provide greater pharmacological precision, the fixed regimen enabled a pragmatic, reproducible evaluation of treatment effects in a real-world setting.

Apart from the observed outcomes, this study has certain inherent limitations, primarily related to its before-and-after design and the absence of a randomised control group, which may limit the generalizability of the findings and preclude causal interpretation of the observed changes. Although no formal statistical adjustment for concomitant therapies (e.g., ANCOVA) was performed, this decision was driven by the limited sample size and the low variability in baseline treatments, which would have made multivariable modelling statistically unreliable and prone to overfitting. The final sample size (*n* = 34) was slightly lower than planned (*n* = 36) due to a low dropout rate (5.6%). Given the minimal attrition, no sensitivity analyses were performed, and post hoc power analysis was not conducted, as it does not provide additional inferential value beyond effect estimates and confidence intervals. Furthermore, the lack of standardised electrodiagnostic data (NCS/EMG) is an additional limitation, as these measures could have provided objective confirmation of nerve involvement and reduced potential expectancy bias associated with patient-reported outcomes. Beyond these methodological considerations, an additional limitation is that complementary biological and mechanistic assessments were outside the scope of the present pragmatic clinical design. Accordingly, no blood biomarker evaluations, including measurements of potential circulating derivatives or metabolites of *Gastrodia elata*, were performed. Future randomised controlled trials should incorporate pharmacokinetic and biomarker analyses to further elucidate mechanistic pathways and strengthen the biological plausibility of the observed clinical associations. Nonetheless, the before-and-after approach allowed for a direct assessment of individual changes from baseline, providing an accurate measure of the supplementation’s effect. Furthermore, the strength of the results, despite methodological constraints, is supported by the use of validated scales commonly employed in clinical practice, such as NPRS, VAS, BCTQ, and BFPS, which have proven sensitivity and reliability in evaluating changes in pain, function, and quality of life.

In interpreting these results, the short-term natural history of radiculopathy and CTS should be considered. Evidence indicates that spontaneous improvement is common within 6–12 weeks in lumbar radiculopathy, with clinically meaningful improvement reported in approximately 20–40% of patients within the first 2–3 months under conservative management [85,86]. Cervical radiculopathy shows a variable but generally favourable course, with clinically meaningful improvement reported in a substantial proportion of patients over several months [87]. In contrast, spontaneous improvement in CTS appears limited; conservative or expectant management may lead to partial symptom relief in approximately 20–30% of mild cases, whereas complete recovery without targeted intervention remains uncommon [88]. In this context, the magnitude of improvement observed over 2 months, particularly the 50–55% reduction in pain intensity (NPRS and VAS), together with improvements in functional outcomes and quality of life, appears clinically meaningful within the limitations of an uncontrolled before–after design. Similar improvements have been reported in observational and clinical studies investigating multicomponent nutraceutical approaches for neuropathic and compressive pain conditions, including CTS, although the available evidence is limited and heterogeneous and does not allow precise quantification of effect size over short-term follow-up [89,90]. However, these comparisons remain indirect, as the absence of a control group precludes separation of treatment effects from placebo response, regression to the mean, and natural symptom fluctuation. Therefore, while the findings are consistent with a potential benefit of the nutraceutical formulation, they should be regarded as exploratory and hypothesis-generating, warranting confirmation in randomised controlled trials.

Despite these limitations, Assonal^®^’s potential for clinically relevant modulations are supported by consistent reductions observed across multiple validated outcome measures in acute pain intensity, as well as improvements in functional outcomes and quality of life. These clinical observations are in line with previously published preclinical data demonstrating that the nutraceutical composition of Assonal^®^ has been shown to promote significant neuroprotective, anti-inflammatory, and antioxidant actions in models of neuropathic pain, as well as by the scientific literature already discussed [20]. These preclinical studies reported decreased pro-inflammatory cytokine levels, attenuation of neuroinflammation, modulation of oxidative stress pathways, and preservation of neuronal integrity.

The translational relevance of our findings is supported by the alignment between mechanistic preclinical evidence and clinical outcomes, with improvement reported by 32 out of 34 participants. Overall, these findings support the hypothesis that multicomponent nutraceutical supplementation was associated with clinically relevant changes in pain-related outcomes and functional performance and may represent a clinically relevant adjunctive strategy in peripheral nerve compression-related pain conditions. No moderate or severe adverse events were reported, and no participants discontinued the intervention. These observations support the need for future randomised controlled trials specifically designed to further investigate these associations, confirm their robustness, and define disease-specific clinical indications.

## 5. Conclusions

In this real-world exploratory study, supplementation with Assonal^®^ was associated with consistent reductions in pain intensity and sensory symptom burden, as well as improvements in functional performance and quality of life in patients with cervical or lumbar radiculopathy and CTS. Although the uncontrolled before–after design does not allow causal inference, the magnitude and consistency of modulations across multiple validated outcome measures provide preliminary clinical evidence supporting the design of adequately powered randomised controlled trials to further investigate these findings and define disease-specific clinical positioning.

## Figures and Tables

**Table 1:** Quantitative composition of Assonal^®^ per daily dose (2 tablets). Values are expressed as mg per daily dose, unless otherwise specified (µg for vitamin B12).

Active Ingredient	Amount per 2 Tablets	Unit
Acetyl L-carnitine	1000	mg
OXADIA^®^ (*Gastrodia elata* Blume extract)	200	mg
Citicoline	250	mg
Vitamin B1	25	mg
Vitamin B2	25	mg
Vitamin B5	18	mg
Vitamin B6	10	mg
Vitamin B12	33	μg
Total (Bioactive ingredients)	1528.033	mg

**Table 2:** Analytical and demographic parameters of the research population. Data that are continuous are presented as means ± standard deviations, while categorical variables are shown as counts (percentages), and ratios as x/y. Abbreviations: AA = Active Arm; BMI = Body Mass Index.

	AA (*n* = 34)
Age (years)	69.50 ± 8.70
BMI (kg/m^2^)	22.32 ± 2.17
Sex (female/male)	15/19
Smoke (habitual smokers)	0
≥3 alcohol units/day	0

## Data Availability

The datasets generated and/or analysed during the current study are not publicly available due to patient privacy restrictions and GDPR compliance. However, anonymised datasets and derived data supporting the findings of this study are available from the corresponding author upon reasonable request, subject to ethical and institutional approval. Statistical code and analytical procedures can also be made available to ensure transparency and reproducibility.

## Abbreviations

The following abbreviations are used in this manuscript:

ALC	Acetyl L-Carnitine.
ANOVA	Analysis of Variance.
BCTQ	Boston Carpal Tunnel Questionnaire.
BMI	Body Mass Index.
BPFS	Back Pain Functional Scale.
CTS	Carpal Tunnel Syndrome.
DRG	Dorsal Root Ganglia.
EQ-5D-5L	EuroQol 5-Dimension 5-Level.
GDPR	General Data Protection Regulation.
GPE	Global Perceived Effect.
HPLC	High-Performance Liquid Chromatography
IASP	International Association for the Study of Pain.
ICF	International Classification of Functioning, Disability and Health.
IL-1β	Interleukin 1 beta.
IL-6	Interleukin 6.
MCID	Minimal Clinically Important Difference.
NF-κB	Nuclear Factor kappa B.
NPRS	Numerical Pain Rating Scale.
NPSI	Neuropathic Pain Symptom Inventory.
NSAIDs	Nonsteroidal Anti-Inflammatory Drugs.
ROS	Reactive Oxygen Species.
SD	Standard Deviation.
SIRT1	Sirtuin 1.
TNF-α	Tumour Necrosis Factor alpha.
VAS	Visual Analogue Scale.

## Appendix A

**Table A1:** Concomitant therapies and protocol-permitted rescue medication (paracetamol) taken during the study period. All concomitant therapies were maintained at stable doses throughout the study period. No initiation or modification of concomitant treatments occurred during follow-up. Paracetamol was permitted as rescue medication and was available to all patients. Its use was on an as-needed basis, infrequent, and non-regular. Reported intake was recorded at follow-up visits to monitor potential confounding effects. No initiation or modification of concomitant treatments occurred during follow-up.

Treatment	Patients, *n* (%)
NSAIDs (stable use)	19 (56%)
Physical therapy	11 (32%)
Gabapentinoids	0
Duloxetine	0
Opioids	0
Acupuncture	0
Paracetamol (rescue medication, available to all patients)	Availability: 34 (100%) Rescue use (as needed): 6 (18%)

**Table A2:** Adverse events during the study period. All adverse events were mild and transient. No moderate or severe adverse events were reported, and no participants discontinued the intervention.

Adverse Event	Patients, *n* (%)	Severity	Onset Timing	Attribution
Gastrointestinal discomfort (mild nausea/bloating)	2 (5.90%)	Mild	Early (week 1–2)	Possibly related
Headache	1 (2.94%)	Mild	Early (week 1)	Possibly related
Transient fatigue	1 (2.94%)	Mild	Mid (week 2–4)	Unrelated
No adverse events reported	30 (88.24%)			

**Table A3:** Exploratory stratified NPRS score across timepoints by pain location and CTS status (mean ± SD).

Subgroup	*n*	T0	T1	T2
Cervical radiculopathy + CTS	7	7.35 ± 1.3	6.05 ± 1.4	3.78 ± 1.2
Cervical radiculopathy without CTS	7	7.25 ± 1.2	5.94 ± 1.3	3.71 ± 1.1
Lumbar radiculopathy + CTS	8	7.26 ± 1.4	5.94 ± 1.5	3.69 ± 1.3
Lumbar radiculopathy without CTS	12	7.05 ± 1.3	5.75 ± 1.4	3.38 ± 1.1
**Total cohort**	**34**	**7.20 ± 1.3**	**5.90 ± 1.5**	**3.60 ± 1.2**

**Table A4:** Exploratory stratified VAS score across timepoints by pain location and CTS status (mean ± SD).

Subgroup	*n*	T0	T1	T2
Cervical radiculopathy + CTS	7	7.40 ± 2.0	6.05 ± 1.6	3.35 ± 1.6
Cervical radiculopathy without CTS	**7**	7.25 ± 2.1	5.95 ± 1.5	3.30 ± 1.7
Lumbar radiculopathy + CTS	8	7.35 ± 2.2	6.00 ± 1.6	3.32 ± 1.8
Lumbar radiculopathy without CTS	12	7.20 ± 2.1	5.97 ± 1.4	3.28 ± 1.7
**Total cohort**	**34**	**7.30 ± 2.1**	**6.00 ± 1.5**	**3.31 ± 1.7**

**Table A5:** Exploratory stratified NPSI score across timepoints by pain location and CTS status (mean ± SD).

Subgroup	*n*	T0	T1	T2
Cervical radiculopathy + CTS	7	85.8 ± 10.1	69.8 ± 12.0	44.8 ± 14.2
Cervical radiculopathy without CTS	**7**	84.9 ± 10.0	68.9 ± 11.8	43.9 ± 14.0
Lumbar radiculopathy + CTS	8	84.6 ± 10.4	68.5 ± 12.5	43.5 ± 14.8
Lumbar radiculopathy without CTS	12	84.0 ± 10.3	67.8 ± 12.3	41.9 ± 14.6
**Total cohort**	**34**	**84.7 ± 10.2**	**68.6 ± 12.2**	**43.3 ± 14.5**

**Table A6:** Exploratory stratified EQ-5D-5L score across timepoints by pain location and CTS status (mean ± SD).

Subgroup	*n*	T0	T1	T2
Cervical radiculopathy + CTS	7	0.50 ± 0.23	0.62 ± 0.19	0.83 ± 0.12
Cervical radiculopathy without CTS	**7**	0.49 ± 0.22	0.63 ± 0.18	0.81 ± 0.11
Lumbar radiculopathy + CTS	8	0.49 ± 0.24	0.62 ± 0.20	0.81 ± 0.13
Lumbar radiculopathy without CTS	12	0.48 ± 0.23	0.61 ± 0.19	0.79 ± 0.12
**Total cohort**	**34**	**0.49 ± 0.23**	**0.62 ± 0.19**	**0.81 ± 0.12**
